# ECONOMIC STUDIES IN BRAZILIAN ORTHOPEDICS: A SCOPING REVIEW

**DOI:** 10.1590/1413-785220263405e301641

**Published:** 2026-08-10

**Authors:** Paulo Vitor Carrijo, Leonardo Addêo Ramos

**Affiliations:** 1 Universidade Federal de Sao Paulo (UNIFESP), Escola Paulista de Medicina, Departamento de Ortopedia e Traumatologia, Disciplina de Medicina Esportiva, Grupo de Traumatologia do Esporte, Sao Paulo, SP, Brazil.

**Keywords:** Costs, Economics, Health, Orthopedics, Review, Brazil, Custo, Economia, Saúde, Ortopedia, Revisão, Brasil

## Abstract

**Objective:**

To identify and analyze economic studies in orthopedics conducted in Brazil, classifying them according to the type and completeness of the analysis.

**Methods:**

Scoping review conducted according to PRISMA-ScR guidelines. Searches were performed in PubMed and BVS, including LILACS, using descriptors related to economic evaluation, orthopedics, and Brazil. Studies conducted exclusively in Brazil were included, with no year restriction.

**Results:**

Ninety-five records were identified, and 13 studies remained after application of the eligibility criteria. Of these, nine were complete economic evaluations and four were incomplete analyses.

**Conclusion:**

There is a scarcity of complete economic evaluations in orthopedics in Brazil. ***Level of Evidence III; Review article.***

## RESUMO

**Objetivo:**

Identificar e analisar os estudos econômicos em ortopedia realizados no Brasil, classificando-os quanto ao tipo e completude da análise.

**Métodos:**

Revisão de escopo conduzida conforme o PRISMA-ScR, com busca nas bases PubMed e BVS (incluindo LILACS), utilizando descritores relacionados à avaliação econômica, ortopedia e Brasil. Foram incluídos estudos realizados exclusivamente no país, sem restrição de ano.

**Resultados:**

Foram identificados 95 registros, restando 13 estudos após aplicação dos critérios de elegibilidade. Desses, 9 foram análises econômicas completas e 4 análises incompletas.

**Conclusão:**

Observa-se escassez de avaliações econômicas completas em ortopedia no Brasil. ***Nível de Evidência III; Artigo de revisão.***

## INTRODUCTION

In recent decades, there has been a global increase in health economic studies. Researchers, industry, and healthcare institutions are increasingly interested in making decisions about which clinical approaches to adopt, based on costs in relation to health outcomes. Given the growing availability of data and the greater ease of obtaining them, it increasingly makes sense to select the best treatment, procedure, or medication through economic analyses.^1^

Within the healthcare setting, orthopedics is a specialty characterized by extensive use of what are known as OPMEs (orthoses, prostheses, and special materials), owing to the surgical treatment of the musculoskeletal system, which requires durable and functional devices that support the maintenance of body structure with early rehabilitation of bones, muscles, tendons, ligaments, and joints. The use of OPMEs in orthopedics drives the industry to produce various types of orthoses, prostheses, plates, and screws – a development that facilitates the emergence of new treatments but, in most cases, is more expensive, thereby increasing the costs of medical care. Consequently, institutional decisions regarding changes in treatment methods and the incorporation of new technologies are challenging and require product-effectiveness analyses to ensure that increasingly limited resources are spent as efficiently as possible, whether in the public sector or in supplemental healthcare. In Brazil, the Unified Health System (SUS) incorporates new treatment methods through cost-effectiveness evaluations analyzed by the National Committee for Health Technology Incorporation (CONITEC), whose purpose is to advise the Ministry of Health on matters related to the incorporation, exclusion, or modification of health technologies in the SUS, as well as on the development or revision of clinical protocols or therapeutic guidelines. CONITEC incorporates new technologies according to the following workflow: the secretariat receives the incorporation request; evaluates the scientific studies submitted (and requests supplementary studies if necessary); reports on the technology, available SUS alternatives, and a critical appraisal of the studies are prepared; CONITEC assesses the reports, issues an opinion, and submits them for public consultation; contributions received are incorporated into the technical report; CONITEC receives the report and ratifies the recommendation.^2^

In the United States (US), which produces one of the largest volumes of scientific articles worldwide, orthopedics still lags behind several other specialties in the production of economic analysis articles, despite the substantial consumption of OPMEs. Notwithstanding the limited number of studies in orthopedics, recent years have seen greater incentives toward production, with the emergence of new guidelines for the field.^1^

In line with what is observed in various Western healthcare systems, the Brazilian healthcare system has been facing considerable impacts driven by high expenditures and limited resources, a situation made all the more serious given that a large share of the population relies exclusively on this public system. Countries with public health systems should be more concerned with economic analyses, which could help allocate these resources more efficiently.^3^ Healthcare managers need to obtain and interpret data that demonstrate the true benefits and risks of health technologies in order to support decision-making and better allocate resources toward the incorporation of technologies that can restructure services.^4^

Given that orthopedics is a surgical specialty that frequently requires the use of OPMEs, and considering the Brazilian context in which 80% of users rely exclusively on a public healthcare system, the objective of this study is to conduct a scoping review of economic studies in orthopedics already carried out in Brazil and indexed in the main databases, in order to analyze the number of articles produced in the country, their types, and the quality of the studies.

## METHODS

The entire scoping review framework was guided by the PRISMA-ScR protocol, a validated checklist for systematic reviews with an extension for scoping reviews.^5^ Due to the scarcity of articles in the field, we determined that a systematic review would not achieve adequate quality or yield a meaningful analysis of the landscape. We opted for a scoping review because it is a study design that maps the gaps in a given area and describes the types of available evidence.^6^

The inclusion criteria required studies to have been conducted exclusively in Brazil, given that this was the setting under evaluation. Articles could be from any year and written in Portuguese, English, or Spanish, provided they assessed a health outcome related to orthopedics. The search aimed to identify all possible economic evaluation articles in orthopedics conducted in Brazil at any point in time, of any type (cost-effectiveness, cost-utility, cost-benefit, cost-minimization) and any level of evidence. All articles underwent review by at least 2 of the authors for inclusion and exclusion criteria, as well as for final classification. At the end of the search, all articles were classified by type of analysis (complete or partial) and by type of economic study.

The search was conducted through PubMed and BVS (which collects data from LILACS, MEDLINE, and other non-indexed databases, including scientific events, theses, and open educational resources from South America and the Caribbean). The search strategies followed the BVS repository of search strategies for health economic evaluation, with terms suggested by the library for this type of research, in English, Spanish, and Portuguese. The terms "Brasil OR Brazil" and "Orthop OR Ortop" were added to define the research setting and the specialty of orthopedics. The search terms used were: "Economics" OR "Cost-Benefit Analysis" OR "Health Care Costs" OR "Costs and Cost Analysis" OR "Cost Allocation" OR "Health Care Rationing" OR "Cost Control" OR "Cost of Illness" OR "Cost Sharing" OR "Direct Service Costs" OR "Drug Costs" OR "Employer Health Costs" OR "Hospital Costs" OR "Health Expenditures" OR "Capital Expenditures" OR "Models, Economic" OR "Economics, Nursing" OR "Economics, Medical" OR "Economics, Pharmaceutical" OR "Economics, Hospital" OR "Economics, Dental" OR "Fees and Charges" OR "Capitation Fee" OR "Fee for Service Plans" OR "Fees, Dental" OR "Fees, Medical" OR "Fees, Pharmaceutical" OR "Prescription Fees" OR "Hospital Charges" OR "Rate Setting and Review" OR "Budgets") OR mj:"/economia" OR ((mh:"Evaluation Study" OR mh:"Evaluation Studies as Topic" OR ti:(evaluaci* OR avaliac* OR evaluation* OR assessment*)) AND ti:(economic* OR economy OR economia*)) OR ti:(economic* OR economia OR cost OR costs OR costly OR costing OR price OR prices OR pricing OR pharmacoeconomic* OR pharmaco-economic* OR "marginal analysis" OR "marginal analyses" OR custo-efetividade OR costo-efectividad OR costo-beneficio OR custo-beneficio OR budget* OR expense OR expenses OR financial OR finance OR finances OR financed OR "value for money" OR "monetary value" OR "monetary value" OR "health expenditures" OR "capital expenditures") OR (ab:(evaluaci* OR avaliac* OR evaluation* OR assessment*) AND ti:(economic* OR economy OR economia*)) OR ab:("análise custo-benefício" OR "cost-benefit analysis" OR "análisis costo-beneficio" OR "análise de custo-benefício" OR "análise de custo-efetividade" OR "custo-efetividade" OR "dados de custo-benefício" OR "costo efectividad" OR "datos de costo-beneficio" OR "cost benefit" OR "cost benefit analysis" OR "cost-effectiveness analysis" OR "cost-utility analysis" OR "costs and benefits" OR "evaluacion economica" OR "avaliacao economica" OR "economic evaluation" OR "marginal analysis" OR "cost benefit analyses" OR "cost benefit data" OR "cost effectiveness analysis" OR "cost utility analysis" OR "cost-benefit analyses" OR "cost-utility analyses" OR "cost-utility analyses" OR "marginal analyses" OR "cost effectiveness" OR "cost-benefit data" OR "value for money" OR "monetary value" OR "monetary value" OR "health expenditures" OR "capital expenditures" OR pharmacoeconomic* OR pharmaco-economic*) OR (ab:(economic* OR economia* OR cost OR costs OR costly OR costing OR price OR prices OR pricing OR budget* OR expense OR expenses OR financial OR finance OR finances OR financed) AND (mh:"Evaluation Study" OR mh:"Evaluation Studies as Topic" OR tw:("evaluation studies" OR "evaluation study" OR "estudos de avaliacao" OR "estudo de avaliacao" OR "estudios de evaluacion" OR "estudio de evaluacion" OR Assessment*))) OR tw:("custos de cuidados de saúde" OR "health care costs" OR "costos de la atención en salud" OR "custos de cuidados médicos" OR "custos de tratamento" OR " costos de la atención médica" OR "costos del tratamiento" OR "health costs" OR "healthcare costs" OR "health care cost" OR "health cost" OR "healthcare cost" OR "medical care cost" OR "treatment cost" OR "medical care costs" OR "treatment costs")) AND (Orthop* OR Ortop*) AND (Brasil* OR Brazil*).^7^

The search was performed on four different dates using the same search terms: June 3, 2022; October 20, 2022; May 9, 2023; and July 24, 2025.

## RESULTS

**Figure 1:**
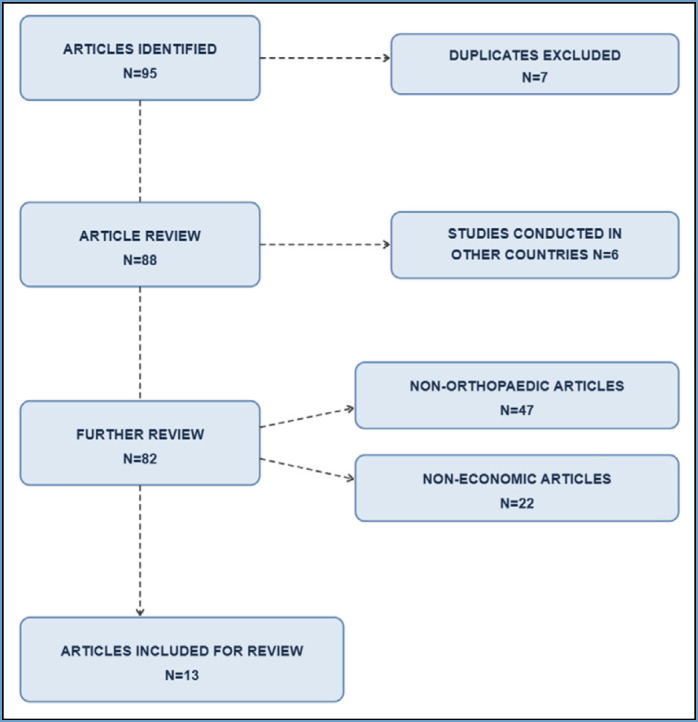
Articles included in the review.

The search retrieved 95 articles, published between 1994 and 2024. The summary of article exclusions and inclusions for the scoping review analysis is presented in the flowchart (Figure 1).

Initially, our search identified 95 articles. We used the BVS-recommended health economic study identifiers to capture as many articles in the field as possible across all time periods, supplemented by the terms "Brasil OR Brazil" and the prefixes "Ortop OR Orthop" to identify articles conducted in Brazil in the specialty of orthopedics. First, seven articles were identified as duplicates and immediately excluded. In the initial review of the remaining 88 articles, six were identified and excluded because they had been conducted in other populations: two from Germany, two from the United States, one from Bangladesh, and one covering all of Latin America. In the final screening, of the remaining 82 articles, 47 were excluded because they were classified as non-orthopedic — that is, articles addressing conditions and treatments from other specialties that did not involve the musculoskeletal system, or even from fields outside medicine such as veterinary or dental articles — and 22 articles were excluded for not being health economic studies, leaving 13 articles that are in fact economic studies in orthopedics conducted in Brazil.

The 13 final articles included in the scoping analysis were classified as either complete or partial analyses, and by type of economic study (cost-effectiveness, cost-utility, cost-benefit, cost-minimization, or simple cost study for partial analyses). The results are presented in Table 1. Of the 13 articles, four were classified as partial analyses, eight as complete economic evaluations that assessed health outcomes, and one was an expert review that considered cost-effectiveness to indicate or include medications in the proposed treatment protocols.

**Table 1:** Articles included in the review.

Article	Year	Analysis type	Study type	Design	Journal	Summary
Fonseca^10^	1999	Partial	Cost	Prospective	Infection control and hospital epidemiology	Cost reduction through an antibiotic prophylaxis program
Ferreira^1^	2011	Complete	Cost-minimization	Prospective	The IOWA orthopaedic Journal	Cost of 2 congenital clubfoot treatment protocols
Loures^2^	2015	Complete	Cost-utility	Retrospective cohort	Revista de saúde pública	Treatment of hip fractures in the elderly
Kaleka^3^	2016	Complete	Cost-effectiveness	Prospective	American Journal of Sports Medicine	Analysis of 2 meniscus measurement methods
Kamamoto^4^	2017	Complete	Cost-effectiveness	RCT	Clinical Science	Analysis of 2 wound treatment methods
Gomes^11^	2017	Partial	Cost	Retrospective	Revista Brasileira de Reumatologia	Cost of rheumatoid arthritis treatment
Passalini^12^	2018	Partial	Cost	Retrospective	Revista da Associação Médica Brasileira	Public insurance expenditure on orthopedic conditions
Souza^5^	2019	Complete	Cost-effectiveness	Retrospective cohort	Revista Brasileira de Ortopedia	Analysis of 2 tibial plateau fixation techniques
Ministério da Saúde^6^	2021	Complete	Cost-minimization	Narrative review	Relatório de recomendação de medicamento - CONITEC	Costs of ibuprofen, naproxen, and diclofenac for musculoskeletal pain
Brito^7^	2022	Complete	Cost-effectiveness	Prospective	Value in Health Regional Issues	Analysis of 3 medications for thromboprophylaxis in arthroplasties
Antonioli^8^	2023	Complete	Cost-effectiveness	Retrospective	BMC Health SR	Cost-effectiveness analysis of alternative treatments to spinal surgery
Veronese^9^	2024	Complete	Cost-effectiveness	Narrative review	Aging clinical Exp R	Narrative review of osteoporosis treatments considering cost-effectiveness
Souza Santos^13^	2024	Partial	Cost	Prospective	J Orthop Sports Phys Ther.	Analysis of costs incurred for musculoskeletal pain treatment in children

Legend: RCT – Randomized controlled trial.

## DISCUSSION

The objective of this study was to evaluate the profile of health economic analysis publications, particularly in orthopedics and traumatology in Brazil, thereby providing an overview of this type of research in our setting. We observed that despite orthopedics being one of the specialties with the highest use of high-cost materials, and despite Brazil's heavy reliance on a public healthcare system, we found only 13 economic studies related to orthopedics conducted in Brazil. According to the ANS (National Supplemental Health Agency), orthopedics was the fifth specialty with the highest volume of outpatient consultations in 2019 (the last year before the pandemic), accounting for more than 14 million outpatient visits in Brazil — this in the supplemental health sector alone, to which only 22% of the population had access through health insurance plans in that year. According to Caetano et al.^8^ in an article from February 2023, over the past 15 years, Brazil has undergone a progressive institutionalization of health technology assessment (HTA), particularly within the public system, highlighting the growing need for the system to draw on economic studies to support healthcare decision-making. The scarcity of studies in this area led the Ministry of Health to establish committees charged with producing scientific evidence to justify policy decisions. However, the fact that these decisions are politically driven rather than grounded in health outcomes and high-quality studies is a concern and underscores the need for the scientific community to engage in this research. Healthcare spending in Brazil is a growing concern, given the challenges facing the healthcare system. According to the Brazilian Institute of Geography and Statistics (IBGE), in 2019 (the last year before the pandemic), total health expenditures represented approximately 9.2% of the country's Gross Domestic Product (GDP). These expenditures encompass public investments, private spending, medication costs, medical consultations, hospital admissions, and other healthcare services.^8-10^

According to the WHO (World Health Organization), public health expenditures vary considerably across nations, accounting for less than 20% of total health costs in high-income countries and up to 66% in low-income countries.^11^ In the US, the projected savings from interventions capable of reducing waste — excluding savings from reduced administrative complexity — ranged from US$191 billion to US$286 billion, representing a potential 25% reduction in total costs, which raises important questions about the implementation of effective measures to eliminate waste. Countries such as Australia, Canada, Sweden, and England (United Kingdom) have already adopted alternative approaches and developed their own guidelines to incentivize the production of economic analyses.^4^ Shrank et al.^12^ (2019), in a more recent study, estimated the magnitude of waste in the American healthcare system to be between US$760 billion and US$935 billion (taking into account waste categories such as overtreatment, care delivery failures, and overpricing, among others).

Another important aspect, beyond the small number of publications, was the types of analyses and studies identified. Health economic analyses can be classified as partial (studies that typically assess only the simple cost of a treatment or a given disease without evaluating health outcomes) or complete (studies that assess cost in relation to some health gain). Complete health economic evaluations are divided into four types: cost-minimization; cost-utility; cost-benefit; and cost-effectiveness. In general, what distinguishes them is the way health outcomes are measured. Cost-minimization studies are those in which identical outcomes are expected from the interventions being compared, and the focus is on comparing total costs. Cost-utility studies assess the effects of interventions on mortality or morbidity, analyzing costs in relation to outcomes such as deaths avoided. Cost-benefit analyses evaluate outcomes in monetary terms, enabling comparisons of expected economic returns relative to investments, thereby facilitating decision-making and efficient resource allocation. Finally, cost-effectiveness studies assess the costs of interventions relative to the best health outcome achieved, enabling concrete clinical practice decisions from these analyses.^13^

We found 4 partial analyses in our review. These are important studies for providing a macro-costing perspective, drawing attention to spending concentrations in a given area, and stimulating the conduct of comprehensive studies on a particular topic; however, they do not allow for high-quality assessment of decision-making. Complete economic evaluations, in contrast, assess health outcomes — most often based on quality of life or preventable events — in relation to cost, thereby enabling evidence-based decision-making by healthcare managers. According to Drummond et al.,^14^ without a complete systematic economic analysis, it is difficult to clearly identify efficient alternatives, increasing the likelihood of wasting time and resources on an inefficient choice. According to Maniadakis et al.^1^, the landscape for economic studies in orthopedics in the US is expected to improve due to recent guidelines and incentives for the production of new research, as is the case in the United Kingdom and Canada.

Among the complete economic evaluations identified in our review, we found two cost-minimization studies, one cost-utility study, and 6 cost-effectiveness studies. The article by Ferreira et al.^15^ was a retrospective study that analyzed the costs of treating congenital clubfoot in a group of 10 children, half of whom were treated using the Kite protocol and the other half using the Ponseti protocol. The study assessed the direct costs of both treatments, and we classified it as a cost-minimization study given that both treatments yield the same outcome according to the literature. The article by Loures et al.^16^, conducted a retrospective decision-tree analysis of the costs of hip fracture treatment, in which patients were divided into 2 groups: early treatment (within four days of trauma) and late treatment, assessing quality of life and mortality outcomes. Kaleka et al.^17^ conducted a prospective study in which they analyzed the most cost-effective imaging method for measuring menisci, comparing magnetic resonance imaging and radiography. Kamamoto et al.^18^ conducted a randomized controlled trial in which they treated 2 groups of patients with complex wounds: one group using a proprietary negative-pressure wound therapy device and another using a low-cost method connected to the hospital vacuum system, which proved to be less expensive and equally effective. Souza et al.^19^ retrospectively studied the cost of two types of implants for tibial osteosynthesis, accounting for device effectiveness by assessing quality of life, functional outcomes, and clinical and radiographic improvement. The Ministry of Health study was a cost-minimization analysis, as a literature review led the authors to conclude that diclofenac, naproxen, and ibuprofen yield the same clinical outcome for chronic musculoskeletal pain, and costs of each were therefore compared for this type of treatment. Brito et al.^20^ conducted a decision-tree analysis to determine which medication is most cost-effective for preventing deep vein thrombosis after knee and hip arthroplasty. Antonioli et al.^21^ also conducted a cost-effectiveness study evaluating 1,088 patients, assessing costs and clinical outcomes in patients with indications for spine surgery who could benefit from alternative treatments. The analysis demonstrated cost savings with clinical effectiveness of USD 6,705 per patient.

A ISPOR^22^ (The Professional Society for Health Economics and Outcomes Research) developed a guideline to promote and standardize high-quality economic studies, known as CHEERS (Consolidated Health Economic Evaluation Reporting Standards), a 24-item checklist. No article mentioned the use of this or any other checklist to guide the economic study, and when we applied CHEERS to the studies, none was able to complete all items on the list.

Cost-effectiveness studies are economic analyses considered appropriate for maximizing health outcomes relative to costs and, as such, are needed in greater numbers to support better and more efficient decision-making; likewise, completing all CHEERS checklist items would help produce studies with stronger methodology.^23^ If we apply rigorous criteria for the selection of cost-effectiveness economic studies in orthopedics, restricting to prospective designs, our review would yield only 3 adequate studies in a country with more than 200 million inhabitants, where approximately 80% rely exclusively on a precarious public health system, in one of the highest-volume specialties.

As a limitation of this type of study, it is difficult to include search terms that capture all relevant articles; however, we believe that our search methodology came close to identifying the totality of indexed studies within the desired scope. Nonetheless, some articles in the field may not have been retrieved because they do not use terms related to the word orthopedics in their texts.

In our search, we found a precarious landscape of health economic studies in orthopedics in Brazil. Very few studies have been conducted overall, and only nine when counting complete analyses. Health economic studies are essential for better evidence-based decision-making in clinical practice, particularly in a resource-constrained setting such as Brazil, where the vast majority of the population depends on the public health system. It is necessary to disseminate this situation and to stimulate the production of new studies so that resources can be deployed more efficiently.

## Data Availability

The data underlying the research text are contained within the manuscript.
